# 

**Published:** 1918-02

**Authors:** 


### ANNOUNCEMENTS

KENTUCKY STATE DENTAL ASSOCIATION will meet in Lexington, June 13 to 15. The general topic for consideration will be an amalgam program which it is hoped will interest all. A cordial invitation is extended to the profession.—W. M. Randall, Secy., 1035 Second St., Louisville.

CONNECTICUT STATE ASSOCIATION will meet at New Haven, April 18 to 20, at the Hotel Taft.—G. S. B. Leonard, Secy.

TEXAS STATE SOCIETY meets April 11-12, in San Antonio.—J. G. Fife, Secy., 736 Wilson Bldg., Dallas.

WEST VIRGINIA STATE SOCIETY meets April 10-12, in Huntington.

A PROFESSIONAL APPEAL. Somewhat over a year ago we asked our wide circle of readers to give sympathetic consideration to an appeal for funds made by "l'Aide Confraternelle," a benevolent organization of our professional confreres of France, the objects of which have a valid claim to our sympathy and support. We regret to say that the appeal brought but a meager response. We again direct attention to this charity, in the hope that a clearer understanding of the matter may bring out a more worthy practical expression of the spirit of fraternal helpfulness which dentistry has always shown in any time of stress or calamity affecting the personnel of its professional body.

Throughout Belgium and the whole area of French territory devastated by the war, practitioners of dentistry, in common with the rest of the population, have had in most instances their every earthly possession swept away. Homeless, dependent families of dentists whose male heads have been mobilized or killed, dentists in civil life dispossessed of means of livelihood and now in dire need are among those for whom the appeal for help is made by l'Aide Confraternelle, which is undertaking the investigation of all appeals of a dental professional origin and is wisely and economically distributing the collected funds in relation thereto. Nor is its larger objective concerned wholly with the alleviation of suffering among dentists and their families during the active war period. By its charter from the French government the organization is to be continued for a period of five years after the conclusion of peace, in order—

(1) To facilitate the return to normal life of all colleagues severely injured by the war, by giving to them or advancing as an honorable debt the necessary means to re-establish themselves; and

(2) In the invaded districts to help in the reconstruction of destroyed homes and in newly equipping dental offices devastated by the enemy, so as to enable dentists to resume their practice.

The officers and management of the organization are representative of the leading dentists of France, with a committee of patrons comprising the most distinguished leaders of the dental profession in that country, who together are chartered as an organization in conformity with the French law. The personnel of l'Aide Confraternelle in itself is ample guarantee of the strength of that body, and of the wisdom, philanthropic spirit, and economy with which its benefactions will be dispensed. Founded in March, 1915, the association has collected up to the present time Fr. 64,968.05, i. e. slightly more than $12,500, contributed almost wholly by French dentists; or, measured by the length of time during which the association has been in active operation, there has been collected less than $5,000 per year, which, considered in relation to the magnitude and importance of the objective for which funds are desperately needed, is meager and wholly inadequate.

The dental profession of America is asked to contribute to a fund to aid destitute and distressed colleagues and their families in the war zone of our allies. Two aspects of this matter present themselves for consideration. The first consideration is a personal and intimate one, obviously because the cry for help comes from our brothers in distress. In every local case when the appeal has been made to the dental profession of America the response has been liberal and prompt; that is to say, when we have understood and realized that the need was one within the limits of our own professional household. But we submit that the present one is precisely such a case. The war is now our war; we share its consequences in common with our allies; our professional household has thus been enlarged by the condition of war so as to embrace every colleague of the Entente nations in the membership of our immediate professional family. We repeat that "these colleagues who have lost all are dependent for relief upon the only enduring and saving human sentiment which stands out as a worthy human characteristic in this maelstrom of world-madness—the spirit of brotherly love." We believe that spirit to be keenly alive among all who struggle for the common blessings of equal freedom and justice to all—blessings which none may rightly enjoy without paying for them in terms of whatever available possession he may have, in service or in substance. We appreciate the fact that appeals for aid necessitated by war conditions are multitudinous, and that each is a pressing one; nevertheless, the meeting of these demands is a condition that war imposes, and none may escape the responsibility of doing his share even though that share shall be the final sacrifice—to help attain the goal. In the present case, in which the appeal is not for a general but for a particular need, a request for aid from dentists to their brother dentists, the opportunity thus afforded becomes a duty as well as a privilege. Prompt action, a little self-denial, and a moment's consideration of the immeasurable good that will result from a whole-hearted response by the dental profession of America to this appeal on behalf of our suffering French and Belgian colleagues and those dear to them, should put the required funds at the disposal of l'Aide Confraternelle without delay.

We ask each reader of this appeal to regard it as personally addressed to him. We ask the editors of all dental periodicals to revoice this appeal in their several publications and to act as receiving stations for contributions, which may be sent direct to the Treasurer of l'Aide Confraternelle, M. Fontañel, 1 Rue Vercingetorix, Paris, France. The *Dental Cosmos* will acknowledge and will so forward any contributions to the fund sent to its publication office addressed to the editor.

---

### TO EVERY AMERICAN DENTIST

BY J. W. BEACH, PRESIDENT, BUFFALO, N. Y.

President Wilson has asked that every resource for winning the war be utilized to the very limit. The dental profession forms one of the greatest resources for making our army efficient. You are an integral part of this great source of help to your country. Will you meet this responsibility as an American citizen should? Of course you will!

### HOW CAN IT BEST BE DONE?

By joining the Preparedness League of American Dentists *now* and assisting in its great work. Ten thousand new members are needed right away. If you are already a member we ask you to get at least five more just as soon as possible. The mouths of the men in our new national army must be made healthy and dentally fit before they go to cantonments and we must help to the limit of our ability.

The Preparedness League of American Dentists is a recognized agency for carrying on this work under the direction of the Surgeon General's office of the War Department, the National Dental Association and the Committee on Dentistry, sub-committee of the Council of National Defense.

There are forty-five thousand dentists in the United States. Six thousand belong to the league and have done the major part of the following work: July 16 to November 3, 1917, 60,946 fillings, 35,909 extractions, 2,233 cleanings, 133 crowns, 184 bridges, 165 plates, 6,891 unclassified operations; total, 111,061. Thousands of operations not listed were performed prior to and since these dates.

If every one of the 45,000 had done his part, what a splendid showing we could have made. It is not too late to become a part of this great work for increasing the fighting power of our army. We know you are with us.

*Don't let the Germans show all the efficiency; let's show what we can do.*

We've got to work together to win this war; do your part by joining the League *today,* and we will give you real, properly-directed work to do. For membership send one dollar ($1.00) payable but once, to the Preparedness League of American Dentists, 131 Allen Street, Buffalo, N. Y. Kindly enclose your business card to avoid mistakes in name and address.

---

### TWO HUNDRED AND FIFTY VACANCIES IN THE DENTAL CORPS

1. The Surgeon General of the army announces that there are, at the present time, approximately 250 vacancies in the Dental Corps, and that examination for the appointment of dental surgeons will be held at various points in the United States on Monday, March 11, 1918.

2. Application blanks and full information concerning these examinations can be procured by addressing "Surgeon General, U. S. Army, Washington, D. C."

3. The Dental Corps is a constituent part of the Medical Corps of the army, and consists of officers in the grades of colonels, lieutenant colonels, majors, captains, and first lieutenants. Appointments therein are made at the rate of one for each 1,000 of the total strength of the regular army, authorized from time to time by law. Law requires that first lieutenants of the Dental Corps shall serve five years in that grade before being promoted, but for the period of the existing emergency this provision has been suspended by act of Congress, and after one year's service as first lieutenant, a dental surgeon is eligible for promotion to the grade of captain, after which promotions are made in order of seniority as vacancies occur in the higher grades.

4. No applicant may under existing law be commissioned in the Dental Corps unless he is between twenty-one and thirty-two years of age, a citizen of the United States, a graduate of a standard dental college, and of good moral character, nor unless he shall pass the usual physical examination required for appointment in the Medical Corps, and a professional examination which shall include tests of skill in practical dentistry and of proficiency in the usual subjects of a standard dental college course. Whether or not the applicant is married has no effect upon his eligibility for the Dental Corps.

5. Application for appointment must be made in writing to the Surgeon General of the army, upon the prescribed blank form. All the interrogatories on the blank must be fully answered. In compliance with the instructions thereon, the application must be accompanied by testimonials, based upon personal acquaintance, from at least two reputable persons, as to the applicant's citizenship, character and habits.

The selection of the candidates is made by the Surgeon General from the applications submitted, and a formal invitation to report for examination to the most convenient examining board in each case will be issued by him.

6. The examinations are conducted under instructions from the Surgeon General and usually last six days. No allowances can be made for the expenses of applicants undergoing examination, whether incurred in travel to and from or during their stay at the place of examination, as public funds are not available for the payment of such expenses.

Each applicant, upon presenting himself to the board, will, prior to his physical examination, be required to submit his diploma as a graduate of a standard dental college. Should he fail to do so, the examination will not proceed.

7. A first lieutenant receives $2,000 per annum; a captain, $2,400 per annum; a major, $3,000 per annum. These salaries are increased by ten per cent. for each period of five years until the maximum of forty per cent. is reached, excepting that the maximum salary of a major is $4,000 a year, and that of a lieutenant colonel and colonel is $375 and $416.66 per month respectively. In addition to their pay proper, they are furnished with a liberal allowance of quarters according to rank, either in kind, or where no suitable government building is available, by commutation. Fuel and light therefor are also provided. When traveling on duty an officer receives mileage for the distance traveled. On change of station he is entitled to transportation of professional books and papers and a reasonable amount of baggage at government expense. Groceries and other articles for their own use may be purchased from the quartermaster at about wholesale cost prices. Dental surgeons are entitled to medical attendance and hospital treatment without charge other than for subsistence.

8. Officers of the Dental Corps are entitled to the privilege of retirement after forty years' service, or at any time for disability incurred in the line of duty. On attaining the age of sixty-four, they are placed on the retired list by operation of law. Retired officers receive three-fourths of the pay of their rank (salary and increase) at the time of retirement.

9. In order to perfect all necessary arrangements for the examination, applications must be in the possession of the Surgeon General at least two weeks before the date of examination. Early attention is therefore enjoined upon intending applicants.

---

### PROPOSED LEGISLATION FOR NAVAL DENTAL CORPS

The National Dental Association, at the New York meeting, approved legislation placing the Naval Dental Corps on an equal status with the Naval Medical Corps, similar to the conditions existing between these two corps in the army, as enacted by Congress, October 6, 1917. The Legislative Committee was instructed to promote this approved legislation at such a time and under such conditions as would seem most favorable. In this connection, and in view of some of the conflicting and discouraging reports regarding what was secured through the Army Dental Corps legislation, it was deemed advisable to wait for this to be officially interpreted before starting legislation for the naval corps. This has just been interpreted to our entire satisfaction, which prompts us to follow the phraseology and general plan of procedure of the army corps legislation. Therefore, the following bill was introduced January 5, 1918, by Senator Lodge (S. No. 3386):

A BILL

To provide for commissioned officers of the Dental Corps of the navy the same rank, pay, promotions and allowances of officers of corresponding grades in the Naval Medical Corps, and for other purposes.

Be it enacted by the Senate and House of Representatives of the United States of America in Congress assembled, That the Dental Corps of the navy shall consist of commissioned officers of the same grades and proportionally distributed among such grades as are now or may be hereafter provided by law for the Medical Corps, who shall have the rank, pay, promotions and allowances of officers of corresponding grades in the Medical Corps, including the right to retirement as in the case of other officers, and there shall be one dental officer for every thousand of the total strength of the navy and Marine Corps authorized from time to time by law: *Provided*, That dental examining boards shall consist of one officer of the Medical Corps and two officers of the Dental Corps: *Provided further*, That immediately following the approval of this act all members of the Dental Corps now in active service shall be recommissioned in the Dental Corps in the grades herein authorized in the order of their seniority and without loss of pay, rank, allowances, or precedence in the navy; and *Provided further*, That nothing in this act shall be construed as in any way affecting the original appointment of officers to the Dental Corps as provided in the "Act approved August twenty-ninth, nineteen hundred and sixteen, making appropriations for the naval service for the fiscal year ending June thirtieth, nineteen hundred and seventeen, and for other purposes;" and *Provided further*, That when ordered to active duty officers of the Dental Reserve Corps shall receive promotion in rank under the same relative conditions and provisions of active service as is provided in this act for the Navy Dental Corps.

It should be distinctly understood that this legislation is entitled to and should receive the liberal support of the profession generally.

To that end we especially and respectfully request that *the officers of all dental societies promptly write their senators and representatives, endorsing this legislation and soliciting their support of same.* It is assumed that all dental societies, at some time, have endorsed this general legislative program and it is therefore suggested that official stationery be used in writing and you be specific in stating that you express the views of your society. Individual letters are very necessary, especially from those who professionally serve or are acquainted with their senators and representatives, and further it is very important that the merits of this legislation be most favorably presented to the members of both the Senate and House Naval Affairs Committees, as committee support is an essential requisite. (A list of these is hereinafter incorporated.) In these letters it can very properly be stated that this proposed legislation is in exact harmony with what was enacted by Congress October 6, 1917, for the Army Dental Corps, and, as a question of justice, the same conditions should be provided for the two branches of the service. This appeal demands your prompt attention and will appreciate it if you will please forward to me such replies as are distinctly favorable or unfavorable, as this makes it possible to keep in touch with the situation in an advantageous way.

The following is a list of the committees above mentioned as published in Congressional Directory, April, 1917:

SENATE NAVAL AFFAIRS COMMITTEE

Benjamin R. Tillman, of South Carolina; Claude A. Swanson, of Virginia; John Walter Smith, of Maryland; Jas. Hamilton Lewis, of Illinois; James D. Phelan, of California; Key Pittman, of Nevada; Thomas J. Walsh, of Montana; Robert F. Broussard, of Louisiana; Peter G. Gerry, of Rhode Island; Park Trammel, of Florida; Boies Penrose, of Pennsylvania; Henry Cabot Lodge, of Massachusetts; William Alden Smith, of Michigan; Carroll S. Page, of Vermont; Miles Poindexter, of Washington; Warren G. Harding, of Ohio; Frederick Hale, of Maine.

HOUSE NAVAL AFFAIRS COMMITTEE

Lemuel P. Padgett, of Tennessee; J. Fred C. Talbott, of Maryland; Albert Estopinal, of Louisiana; Daniel Riordan, of New York; Walter L. Hensley, of Missouri; John R. Connelly, of Kansas; William B. Oliver, of Alabama; William W. Venable, of Mississippi; Carl Vinson, of Georgia; Adam B. Littlepage, of West Virginia; James C. Wilson, of Texas; Thomas S. Butler, of Pennsylvania; William J. Browning, of New Jersey; John R. Farr, of Pennsylvania; Fred A. Britten, of Illinois; Patrick H. Kelley, of Michigan; Sydney E. Mudd, of Maryland.

---

### INTERPRETATION OF ARMY DENTAL CORPS LEGISLATION

Members of the dental profession will probably be interested in knowing that the legislation enacted October 6, 1917, has finally been satisfactorily interpreted and that the members of the Regular Army Dental Corps have received their promotion, subject to the required examination incident to promotion. This interpretation gives us all for which we have contended, and harmonizes very accurately with the views presented in our report at the New York meeting. In this report we incorporated the estimates of the Army and Navy Register, relative to the number in the various grades, but those figures were under stated, since we get twelve colonels instead of nine, twenty lieutenant colonels instead of sixteen, and eighty-seven majors instead of seventy-one. In addition there will be something more than one hundered first lieutenants, of which a proportionate number will be promoted to the grade of captain as soon as they have completed one year's service. This promotion on the basis of one year's service is the result of the emergency legislation for the term of the war and was authorized by the provisions of H. R. 4897, to which bill we offered our dental amendment. In view of the fact that the Dental Corps is placed on an exact status with the Medical Corps, our corps naturally gets the benefit of this emergency legislation.

While no specific mention was made of the Dental Reserve Corps in our recent legislation, my contention has always been that the legislation for the regular corps automatically provided the authority for the necessary modification of the regulation relative to the Officers' Reserve Corps to place the Dental Reserve Corps on an equal status with the Medical Reserve Corps. This position has been thoroughly verified by the official interpretation and in the future whatever applies to the Medical Reserve Corps will apply in like manner to the Dental Reserve Corps.

In connection with this legislation, I received hundreds of congratulatory messages, consisting of letters, resolutions, telegrams and cablegrams. These were received at a time when it was impossible to give them anything like the prompt individual attention they merited. Then followed weeks of conflicting reports, but now that this has been officially and satisfactorily interpreted, I take this belated and public method of kindly thanking all for their generous expressions. Fraternally, Homer C. Brown, Chairman Legislative Committee, N. D. A., 609 Hartman Building, Columbus, Ohio, January 15, 1918.

